# NMD abnormalities during brain development in the *Fmr1*-knockout mouse model of fragile X syndrome

**DOI:** 10.1186/s13059-021-02530-9

**Published:** 2021-11-16

**Authors:** Tatsuaki Kurosaki, Hitomi Sakano, Christoph Pröschel, Jason Wheeler, Alexander Hewko, Lynne E. Maquat

**Affiliations:** 1 Department of Biochemistry and Biophysics, School of Medicine and Dentistry, University of Rochester, Rochester, NY 14642 USA; 2 Center for RNA Biology, University of Rochester, Rochester, NY 14642 USA; 3 Department of Otolaryngology, School of Medicine and Dentistry, University of Rochester, Rochester, NY 14642 USA; 4 Department of Biomedical Genetics, School of Medicine and Dentistry, University of Rochester, Rochester, NY 14642 USA; 5 Stem Cell and Regenerative Medicine Institute, School of Medicine and Dentistry, University of Rochester, Rochester, NY 14642 USA

**Keywords:** Nonsense-mediated mRNA decay (NMD), Fragile X protein (FMRP), Upframeshift protein 1 (UPF1), *Fmr1*-KO mouse, Mouse brain development, Cortex, Hippocampus, Cerebellum, Fragile X syndrome

## Abstract

**Background:**

Fragile X syndrome (FXS) is an intellectual disability attributable to loss of fragile X protein (FMRP). We previously demonstrated that FMRP binds mRNAs targeted for nonsense-mediated mRNA decay (NMD) and that FMRP loss results in hyperactivated NMD and inhibition of neuronal differentiation in human stem cells.

**Results:**

We show here that NMD is hyperactivated during the development of the cerebral cortex, hippocampus, and cerebellum in the *Fmr1*-knockout (KO) mouse during embryonic and early postnatal periods. Our findings demonstrate that NMD regulates many neuronal mRNAs that are important for mouse brain development.

**Conclusions:**

We reveal the abnormal regulation of these mRNAs in the *Fmr1*-KO mouse, a model of FXS, and highlight the importance of early intervention.

**Supplementary Information:**

The online version contains supplementary material available at 10.1186/s13059-021-02530-9.

## Background

A deficiency in the RNA-binding protein FMRP is a hallmark of FXS, the leading single-gene cause of autism [1–3]. Given that FMRP is estimated to bind only ~ 4–5% of mRNAs in human fetal brain [4] and mouse brain [5], it was unexpected that human neuronal mRNAs that co-immunoprecipitate with FMRP are highly enriched in mRNAs that are targeted for NMD (e.g., there is an average ~ 2-fold enrichment of FMRP with NMD targets relative to other cellular mRNAs) [6]. In explanation of this, the binding of FMRP to NMD targets is facilitated by another RNA-binding protein, the key NMD factor upframeshift 1 (UPF1), which is preferentially bound to NMD targets [7] and directly binds FMRP [6]. Small molecules that inhibit NMD were found to normalize the differentiation of induced pluripotent stem cells (iPSCs) derived from FXS-patient fibroblasts to neurons, consistent with the importance of proteins encoded by human NMD targets for proper neuronal-cell differentiation [6].

The *Fmr*1-KO mouse, which lacks FMRP, is a well-studied model of FXS [8–10] that offers the opportunity to study brain development in the absence of FMRP. To determine if FMRP loss in mouse also results in hyperactivated NMD, we first identify NMD targets in mouse Neuro-2a (N2A) neuroblastoma cells, which are readily amenable to transfection and biochemical analyses. We then demonstrate that NMD is indeed hyperactivated in *Fmr*1-KO mouse brain in the perinatal period using two distinct quantitative assays to measure NMD efficiency: the levels of activated, i.e., phosphorylated, UPF1 (p-UPF1), and the levels of NMD targets (normalized to the level of pre-mRNA from which each derived). Our findings map the developmental window of NMD hyperactivation in the *Fmr1*-KO mouse model of FXS and provide a foundation to further examine the effect of hyperactivated NMD on neuronal differentiation in the cortex, hippocampus, and cerebellum, as well as a rationale for early intervention in FXS using NMD inhibitors.

## Results and discussion

**Fig. 1:**
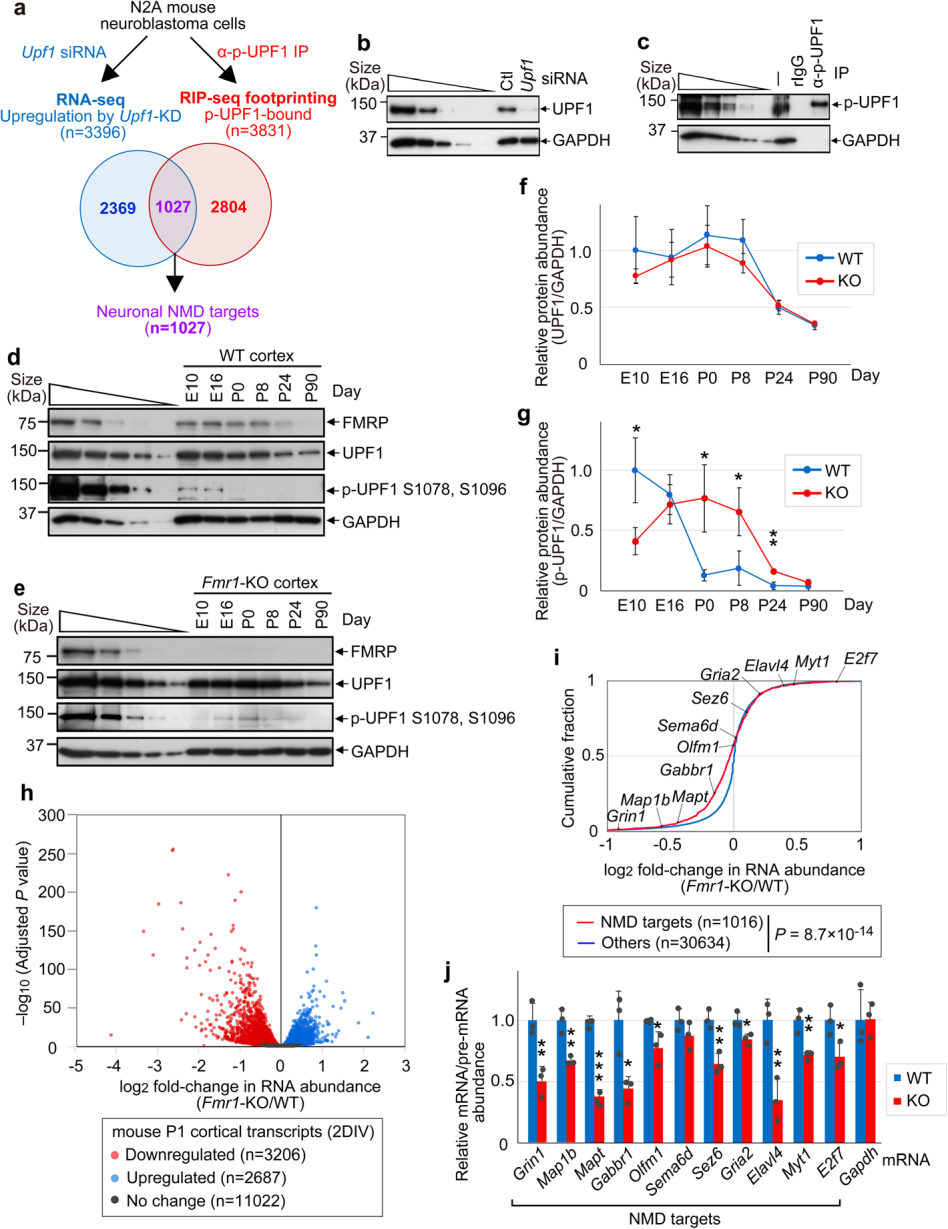
NMD is hyperactivated during mouse cortex development. **a** Venn diagram defining mouse N2A-cell NMD targets based on their upregulation by *Upf1* siRNA (UPF1-KD) relative to control siRNA (log_2_ fold-change > 0, adjusted *P* value < 0.05) as determined by RNA-sequencing (RNA-seq), denoted in blue, and their increased immunoprecipitation (IP) using antibody (α) to phosphorylated UPF1 (p-UPF1) and RNA IP (RIP)-seq footprinting relative to size-matched input Neuro2A(N2A) RNA (log_2_ fold-change > 1, adjusted *P* value < 0.05), shown in red. *n*, number of transcripts. **b** Western blots of lysates of N2A cells cultured with either control (Ctl) siRNA or *Upf1* siRNA used in **a**. Results represent three independently performed experiments. Here and elsewhere, leftmost lanes under the wedge represent 3-fold serial dilutions to show that results are within the linear range of analysis. **c** As in **b**, but before (−) or after IP using α-p-UPF1 or, as a control, rabbit IgG (rIgG). Results represent three independent experiments. **d** Western blot of cortex from wild-type (WT) mice at the denoted day of embryonic (E) or postnatal (P) development using the specified antibody. Results represent three independent experiments. **e** As in **d**, but using *Fmr1*-knockout (KO) mouse cortex. Results represent three independent experiments. **f** Line plot of the ratio of UPF1 normalized to GAPDH as quantitated from **d** and **e**. Means ± S.D., where *n* = 3 (WT) and 3 (*Fmr1*-KO). **g** As in **f**, but for the ratio of p-UPF1 normalized to GAPDH. Means ± S.D., where *n* = 3 (WT) and 3 (*Fmr1*-KO). (*)*P* < 0.05 or (**)*P* < 0.01 compares *Fmr1*-KO cells relative to WT cells (two-sided unpaired t-test). **h** Volcano plot of RNA-seq data deriving from 2-day cultures of neurons from WT or *Fmr1*-KO mouse P1 cortex. All results derive from independently derived samples in triplicate. **i** Using data from **h**, cumulative fraction analysis, where the category “Others” consists of cellular transcripts not defined in **a** as NMD targets. *n*, number of transcripts. *P* values were calculated by the two-sided Wilcoxon rank-sum test. **j** Histogram representation of RT-qPCR demonstrating that NMD is hyperactivated in cultured neurons from the P1 cortex of *Fmr1*-KO mice. The level of each NMD target was normalized to the level of the pre-mRNA from which it derives, and the normalized level in WT mice is defined as 1. Means with S.D., where *n* = 3 (WT) and 3 (*Fmr1*-KO). (*)*P* < 0.05, (**)*P* < 0.01 or (***)*P* < 0.001 compares *Fmr1*-KO cells relative to WT cells (two-sided unpaired *t* test)

We used our established transcriptome-wide RNA sequencing (RNA-seq) methodologies [6] to identify high-confidence NMD targets in mouse N2A neuroblastoma cells, an abundant and biochemically accessible homogeneous source of mouse neural cells. Through parallel analyses of RNA-seq upon UPF1-knockdown (KD), and RNA immunoprecipitation (RIP-seq) footprinting of p-UPF1-bound RNAs (Fig. 1a–c), we identified 1027 high-confidence neuronal NMD targets (Fig. 1a and Additional file 1: Table S1; hereafter referred to as NMD targets). This is consistent with findings for human SH-SY5Y neuroblastoma and other mammalian cells that ~ 5–10% of protein-encoding cellular genes produce NMD targets [6, 7, 11].

The neuronal NMD targets identified here are expressed throughout the mouse brain (Additional file 2: Fig. S1a). In particular, > 50% (527/1027) of NMD targets defined for N2A cells produce proteins in the cerebral cortex, which regulates motor function and aspects of cognition that include attention and language. The functional significance that 421 NMD targets defined for N2A cells are expressed in the hippocampus (Additional file 2: Fig. S1a) is exemplified by the finding that inhibiting NMD after a prolonged seizure using the small molecule NMDI-14 reduced subsequent spontaneous seizures [12]. Among them, 407 of the 421 NMD targets expressed in the hippocampus are also expressed in the cerebral cortex (Additional file 2: Fig. S1a). Gene Ontology (GO) term enrichment analysis for biological process followed by network analysis revealed that N2A-cell NMD targets are indeed significantly enriched to encode proteins that function in neuronal signaling processes such as neuronal synaptic organization, regulation of exocytosis, and neurotransmitter secretion (Additional file 2: Fig. S1b).

To test for possible differences in the efficiency of NMD in *Fmr1*-KO relative to wild-type (WT) mice, we first examined protein isolated from the cerebral cortex, hippocampus, and cerebellum. The cerebral cortex was of particular interest given its overall high abundance of FMRP [13], and our ability to isolate cortical tissue as early as embryonic day 10 (E10) [14]. While the level of UPF1 in WT mice relative to *Fmr1*-KO mice remained comparable at all developmental stages that were assayed (Fig. 1d, e), the level of phosphorylated UPF1 (p-UPF1), which can be used as a proxy for NMD efficiency [6, 7, 15], showed developmentally delayed expression and persistently elevated levels in the perinatal period in *Fmr1*-KO mice. NMD is most active at E10 and E16 in WT mice, during which time p-UPF1 levels are just starting to increase in *Fmr1*-KO mice. NMD efficiency then precipitously drops by P0 in WT mice but remains abnormally high through P24 in *Fmr1*-KO mice (Fig. 1f, g). Given these findings, and that NMD has been shown to promote the stem cell state of mouse neuronal stem cells derived from E14.5 brains grown as neurospheres and to decrease in efficiency once these cells were differentiated by withdrawing growth factors [16], it follows that neuronal cell differentiation would be abnormally delayed in *Fmr1*-KO mice.

Consistent with FMRP loss hyperactivating NMD during development, RT-qPCR demonstrated that the abundance of each of three NMD targets (*Grin1*, *Map1b*, and *Mapt*) in *Fmr1-KO* relative to WT mice were abnormally low in P0 and P8 cortex (Additional file 2: Fig. S1c). Moreover, RNA-seq of cortical neurons deriving from 2-day cultures of P1 cortex (Fig. 1h) revealed that the levels of NMD targets defined here (Fig. 1a and Additional file 1: Table S1) were generally and significantly (*P* = 8.7 × 10^−14^) decreased in *Fmr1*-KO relative to WT mice (Fig. 1h, i), concomitantly with an increased abundance of p-UPF1 (Additional file 2: Fig. S1d). Our rationale for performing RNA-seq using P1 cortex cultured for 2 days in vitro rather than using cortex per se was to reduce the cell-type heterogeneity that typifies cortex.

GO term enrichment analysis for biological process revealed that the downregulated transcripts encode proteins typically functioning in various synaptic signaling pathways (Additional file 2: Fig. S1e, and Additional file 3: Table S2). However, it should be noted that utilizing NMD targets that we established for N2A neuroblastoma cells to define those expressed in 2-day cultures of P1 cortex would overlook NMD targets expressed in 2-day cultures but not in N2A cells. As an additional assay of NMD efficiency, RT-qPCR was used to normalize the level of 11 NMD targets in 2-day cultures of P1 cortex from WT and *Fmr1*-KO mice to the level of pre-mRNA from which each derives, the latter of which controls for changes in NMD target abundance that could be due to changes in mRNA synthesis rather than hyperactivated NMD in *Fmr1*-KO mice. Results indicated that *Fmr1*-KO indeed hyperactivates NMD (Fig. 1j). Of these mRNAs, *Gabbr1* mRNA was previously deemed to be an NMD target given its upregulation in *Upf1* siRNA-treated mouse embryonic stem cells [17]. Notably, these NMD targets, i.e., *E2f7*, *Myt1*, *Elavl4*, *Gria2*, and *Sez6* mRNAs, include those upregulated in abundance, as measured either by RNA-seq (Fig. 1i) or RT-qPCR (data not shown), prior to normalizing to the even larger upregulation in the production of their pre-mRNAs (Fig. 1j), i.e., mRNAs whose enhanced NMD is masked by their increased synthesis.

**Fig. 2:**
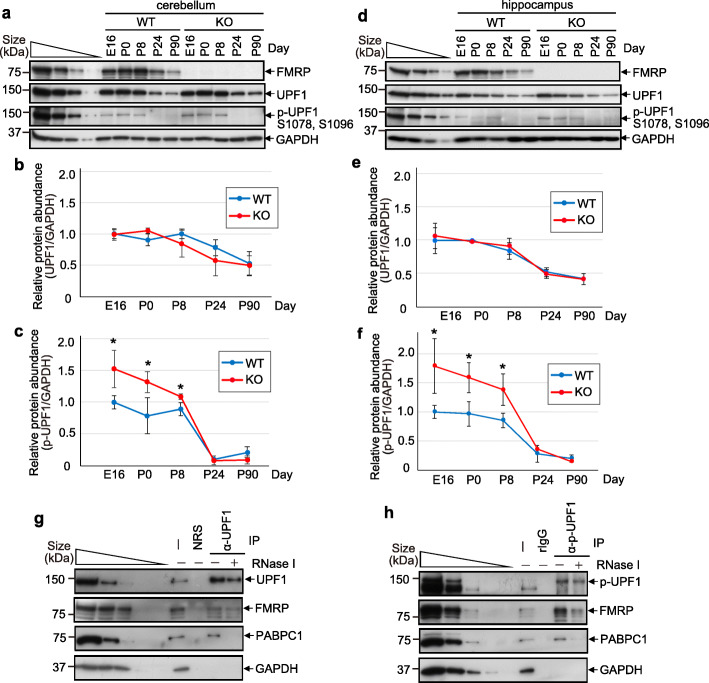
NMD is hyperactivated during mouse cerebellum and hippocampus development in the *Fmr1*-KO mouse, and FMRP co-immunoprecipitates with UPF1 and p-UPF1 in P0 cortex of the WT mouse. **a** As in Fig. 1d, but for WT and *Fmr1*-KO cerebellum. Representative of three independently performed experiments. **b** As in Fig. 1f, but for WT and *Fmr1*-KO cerebellum. Means ± S.D., where *n* = 3 (WT) and 3 (*Fmr1*-KO). **c** As in Fig. 1 g, but for WT and *Fmr1*-KO cerebellum. Means ± S.D., where *n* = 3 (WT) or 3 (*Fmr1*-KO). (*)*P* < 0.05 compares *Fmr1*-KO cells relative to WT cells (two-sided unpaired *t* test). **d** As in **a**, but for the hippocampus. **e** As in **b**, but for the hippocampus. **f** As in **c**, but for the hippocampus. Means ± S.D., where *n* = 3 (WT) and 3 (*Fmr1*-KO). (*)*P* < 0.05 compares *Fmr1*-KO cells relative to WT cells (two-sided unpaired *t* test). **g** Western blots of lysates of WT P0 cortex before (−) or after IP with (+) or without (−) RNase I using anti(α)-UPF1 or, as a negative control, normal rabbit serum (NRS). Representative of two independently performed experiments. **h** As in **g**, but using α-p-UPF1 and, as a negative control rabbit IgG (rIgG) in IPs. Representative of two independently performed experiments

We additionally analyzed NMD efficiency in the hippocampus and cerebellum of WT and *Fmr1*-KO mice, but beginning on E16 rather than E10 since both regions are underdeveloped at E10. Quantification of the level of UPF1 or p-UPF1 in the cerebellum (Fig. 2a–c) and hippocampus (Fig. 2d–f) demonstrated that the level of UPF1 was similar for WT and *Fmr1*-KO mice at all developmental stages analyzed, but the level of p-UPF1 in *Fmr1*-KO mice was abnormally elevated from E16 through P8 before returning to normal by P24 (Fig. 2a–f).

The mechanism by which NMD is hyperactivated in human iPSCs involves a direct interaction between FMRP and UPF1; this interaction facilitates the recruitment and/or stabilizes the binding of FMRP to NMD targets via its association with UPF1 and p-UPF1 [6]. To determine if FMRP and UPF1 function analogously in vivo, UPF1 and p-UPF1 were immunoprecipitated from lysates of WT P0 cortex in the absence or presence of RNase I. FMRP was detected in each immunoprecipitation (IP) in a partially RNase I-resistant manner (Fig. 2g, h), providing an indication that the direct interaction between the two proteins observed in human cells is conserved in mouse. The same result was evident for P0 cerebellum (data not shown). This makes sense given that the interspecies amino-acid identities of FMRP and of UPF1 are 96% and 98.5%, respectively (Additional file 2: Fig. S2a and b). As controls, the co-IP of the poly(A)-binding protein PABPC1 was sensitive to RNase I treatment, GAPDH failed to co-immunoprecipitate with either UPF1 or p-UPF1, and no protein was immunoprecipitated in control IPs that used normal rabbit serum or rabbit IgG (Fig. 2g, h).

Consistent with an interaction of FMRP with UPF1 and p-UPF1 (Fig. 2g, h) [6], we find that p-UPF1 and FMRP are co-expressed in cells of the developing cortex, hippocampus, and cerebellum (Additional file 2: Fig. S2c). At P0 in the mouse, we find that p-UPF1 is widely expressed in the developing cortex, and particularly in the cortical plate (CP) and subventricular zone (SVZ). As previously reported [13, 18–21], FMRP is also widely expressed in the developing cortex, including the CP. In the hippocampus, p-UPF1 and FMRP are highly co-expressed in the granule and molecular layers of Cornus Ammon regions 1–3 (CA1-3), and expression of FMRP and p-UPF1 in the cerebellum overlaps extensively in the molecular cell layer (Additional file 2: Fig. S2c) [13, 22]. Thus, while p-UPF1 and FMPR staining can also be observed in germinal zones such as the ventricular zone of the cortex and dentate gyrus of the hippocampus, the highest level of co-expression was found in areas of differentiating neuronal-cell populations, i.e., the CP and molecular layer.

At the subcellular level, FMRP, UPF1, and p-UPF1 were co-localized in both the soma and the neurites of cortical neurons in vivo and in vitro (Additional file 2: Fig. S2d and e, respectively) by confocal imaging. Since the co-localization of FMRP and p-UPF1 is known to also occur on translating NMD targets in human cells [6], our localization of both proteins to neurites (Additional file 2: S2d and e) is consistent with the recent report that FMRP promotes RNA localization to neuronal projections in mouse neuronal catecholaminergic CAD cells [23].

## Conclusions

We show here that NMD controls the production of proteins throughout the mouse brain (Additional file 2: Fig. S1a) and, by doing so, regulates neuron differentiation, including the development of neuronal processes, synapse organization, and neurotransmitter secretion (Additional file 2: Fig. S1b). These results are consistent with wide co-expression of FMRP and p-UPF1 in the developing brain (Additional file 2: Fig S2c and d). Our findings reinforce data illustrating the importance of properly regulated NMD to proper neurogenesis in both humans and mice [6, 16, 24–27], and how loss of FMRP results in misregulated NMD both in utero and after birth (Figs. 1 and 2).

Prior to this work, connections between FMRP and the regulation of NMD efficiency in a way that is essential for proper neurogenesis in the mouse were largely anecdotal. As one example that can be inferred from studies using rats, the NMD of *Arc* mRNA, a dendritic mRNA, has been shown in rats to depend on eIF4A3, a constituent of the exon-junction complex that is deposited on newly made mRNAs as a consequence of pre-mRNA splicing [28]. The same study demonstrated that eIF4A3-KD in cultured rat neurons not only inhibits the NMD of *Arc* mRNA but also leads to increased miniature excitatory postsynaptic currents, consistent with an increase in the synaptic abundance of glutamate receptor 1 (GluR1), which is a type of α-amino-3-hydroxy-5-methyl-4-isoxazolepropionic acid (AMPA) receptor [28]. Notably, *Arc* mRNA translation is strictly regulated by FMRP dependent on group 1 metabotropic glutamate receptor (mGluR) stimulation and long-term depression of excitatory synaptic transmission in mouse and rat hippocampal neurons [29].

As another example, loss of FMRP in primary mouse hippocampal neurons results in destabilization of postsynaptic density 95 (*Psd95*) mRNA [30], which binds FMRP and encodes a protein essential for synaptic plasticity and learning [31]. The FMRP-mediated stabilization of *Psd95* mRNA is promoted by mGluR activation [30], and the level of *Psd95* mRNA is post-transcriptionally regulated by alternative splicing coupled to NMD during the early developmental stage of mouse cortical neurons [32], providing another molecular link between FMRP and NMD.

To further complicate mechanistic differences between WT and *Fmr1*-KO mice, FMRP has been shown to regulate many aspects of gene expression beyond mRNA translation, including chromatin modification, RNA synthesis and processing, adenosine deaminases acting on RNA, and microRNA associations with RNA-induced silencing complexes [33].

Our finding that NMD is hyperactivated at particular developmental stages in utero and postnatally in *Fmr1*-KO mice suggests that small molecule inhibitors of NMD, which have already shown efficacy when administered to mice to rectify neuronal-cell metabolism [12, 34], should be tested as a means of restoring gene expression and behavioral and cognitive functions [35, 36]. There have already been numerous studies focusing on potential treatments for FXS by targeting the aberrant synaptic signaling pathways. Despite promising results with mGluR5 inhibition using the *Fmr1*-KO mouse [37–39], results from large clinical trials in patients age 12 and older have been disappointing [40]. There are currently several other ongoing trials that involve pharmacological intervention of neuronal transmission to treat patients, only few of whom are younger than age 5 (https://clinicaltrials.gov/). Our results indicate that a very early developmental period, essentially at and soon after the time of birth, may define the window of opportunity during which an alternative therapeutic pathway, i.e., inhibition of NMD, may be helpful. Such an approach would require earlier diagnoses that, given the prevalence of this disease, may not be an unreasonable goal.

## Methods

### Animals

Wild-type (WT) and *Fmr1*-knock-out (KO) mice in the C57BL/6 J background (The Jackson Laboratory) were used in this study. All animal work was performed in the University of Rochester Medical Center (URMC) vivarium and according to the protocol approved by the University Committee on Animal Resources (UCAR-2018-027).

### Mouse brain harvesting

For fresh brain-tissue collection, mice were euthanized by carbon dioxide anesthesia, the brain was excised from the skull, and specific regions were removed and quickly frozen on dry ice. For perfused tissues, mice were anesthetized with ketamine (100 mg/kg)/xylazine (10 mg/kg) intraperitoneal injection and perfused with phosphate buffered saline (PBS) followed by 4% paraformaldehyde (PFA) in PBS. The brains were removed from the skull and post-fixed in 4% PFA for 2 h at 4 °C and dehydrated in 30% sucrose/PBS for 2 days before embedding in Tissue-Tek OCT Compound (Sakura Finetek USA). Cryosections (14 μm) were obtained using a Leica CM1860UV at the URMC Histology, Biochemistry, and Molecular Imaging core facility.

### Cell cultures and transfections

Commercially obtained N2A mouse neuroblastoma cells (ATCC, CCL-131), which were authenticated by ATCC and free of mycoplasma, were cultured in Dulbecco’s modified Eagle’s medium/Nutrient Mixture F-12 (DMEM/F-12, Thermo Fisher Scientific) supplemented with 10% fetal bovine serum (VWR). Cells (0.3 × 10^6^/6-well plate) were transiently transfected with 60 pmol of Ambion Silencer Negative Control #1 siRNA (Thermo Fisher Scientific) or *Upf1* siRNA (Additional file 3: Table S2) using the TransIT-X2 Dynamic Delivery System (Mirus Bio). For p-UPF1 RIP-seq, N2A cells were treated with 200 nM okadaic acid (Sigma) for 2 h prior to cell lysis and immunoprecipitation.

Cerebral cortical tissues were removed from WT or *Fmr1*-KO P1 pups and dissociated using papain protease (Worthington) and mechanical trituration. Dissociated cells were filtered using 40 μm cell strainers (BD Falcon) to remove blood vessels and tissue clumps, then immune-depleted of ACSA2^+^ astrocytes [41] and myelin using anti-ACSA-2 and anti-myelin microbeads (Miltenyi Biotech), respectively, and were then plated on 5 ng/ml laminin/poly-L lysine substrate (Thermo Fisher Scientific) at 4.2 × 10^4^ cells/cm^2^ and cultured for 2 days in Neurobasal/B27 Plus Medium (Thermo Fisher Scientific) supplemented with 1× penicillin-streptomycin solution (Thermo Fisher Scientific).

### Immunoprecipitations

Immunoprecipitations (IPs) were performed as described previously [6, 42] in the presence or absence of 1 U/μl of RNase І (Thermo Fisher Scientific, AM2294) using the following antibodies: anti-UPF1 [43], anti-p-UPF1 S1116 (MilliporeSigma, 07-1016) or, as a negative control, either normal rabbit serum (NRS, Sigma-Aldrich) or rabbit IgG (rIgG, Sigma-Aldrich).

### Protein and RNA preparations

Cells and tissues were lysed using cycles of pipetting, vortexing, and incubating on ice (in that order) over a 10-min period in Hypotonic Gentle Lysis Buffer [10 mM Tris (pH 7.4), 10 mM NaCl, 10 mM EDTA, 0.5% w/w Triton X-100 (Thermo Fisher Scientific)] supplemented with 1× Halt Protease and Phosphatase Inhibitor Cocktail (Thermo Fisher Scientific). After the addition of NaCl to 150 mM, lysates were centrifuged at 15,000×*g* for 10 min and used in protein analyses. RNA was extracted and purified using TRIzol reagent following the manufacturer’s instructions (Thermo Fisher Scientific).

### Western blotting

Western blotting was performed as described previously [6, 7, 44] using the following antibodies: anti-UPF1 [43]; anti-p-UPF1 S1078, S1096 [7, 45]; anti-p-UPF1 S1116 (Millipore Sigma, 07-1016); anti-FMRP (Abcam, ab17722); anti-GAPDH (Cell Signaling, 2118S); and anti-PABPC1 (Abcam, ab21060). Uncropped western blots are included as Additional file 5.

### RT-qPCR

RT-qPCR was undertaken as described previously [6, 7]. PCR primer pairs are provided in Additional file 3: Table S2.

### Sequence alignment and sequence similarity analyses

Sequence alignment was performed using BioEdit ver. 7.0.9.0 and CLUSTAL X ver. 2.0, and sequence similarity was undertaken using the BLOSUM62 matrix.

### RNA library preparation for RNA-seq and RIP-seq

For RNA-seq library preparation using RNA from N2A cells, or P1 cortical cells after culturing for 2 days, RNA concentration and quality were evaluated using a NanoDrop 1000 spectrophotometer (Thermo Fisher Scientific) and a Bioanalyzer 2100 (Agilent), respectively. RNA-seq libraries were constructed using the TruSeq Stranded mRNA Library Prep Kit (Illumina) and 200 ng of RNA following the manufacturer’s instructions. The quantity and quality of libraries were measured using a Qubit 4 Fluorometer (Thermo Fisher Scientific) and a 5300 Fragment Analyzer System (Agilent), respectively. Libraries were sequenced using a NovaSeq 6000 SP Flowcell (Illumina), generating single-end reads of 100 nucleotides with at least 25 million raw reads per sample.

For RIP-seq library preparation, total or immunoprecipitated N2A-cell RNA was cleaved using RNase I (Thermo Fisher Scientific), and 25–50-nt fragments were size-selected after electrophoresis in 12% polyacrylamide containing 6 M urea [42]. Fragment concentration and quality were assessed using a NanoDrop 1000 Spectrophotometer and Bioanalyzer 2100, respectively. Sequencing libraries were generated using the NEBNext Small RNA Library Prep Kit (New England Biolabs) and 100 ng of fragmented total or immunoprecipitated N2A-cell RNA, which was ligated to 3′- and 5′-adaptors as specified by the manufacturer. cDNA was synthesized, and libraries were generated using 12 PCR cycles. Library quantity and quality were assessed as described above for RNA-seq. Library fragments between 125−200 basepairs (considering that the adaptors added 120 basepairs) were size-selected using PippinHT (Sage Science) after electrophoresis in a 3% agarose gel. Sequencing employed NextSeq 550 High Output Flowcell (Illumina), generating single-end reads of 50 nucleotides.

### Computational analyses of RNA-seq and RIP-seq data

Raw reads generated from the Illumina basecalls were demultiplexed using bcl2fastq version 2.19.1. Quality filtering and adapter removal were performed using FastP version 0.20.0 or 0.20.1 with parameters “–length_required 35 –cut_front_window_size 1 –cut_front_mean_quality 13 –cut_front –cut_tail_window_size 1 –cut_tail_mean_quality 13 –cut_tail –w 8 -y –r -j”. Cleaned reads were then mapped to the *Mus musculus* reference genome (GRCm38.p6 + Gencode-M22 Annotation) using STAR_2.7.0f or 2.7.6a and parameters “–twopass Mode Basic –runMode alignReads –outSAMtype BAM Unsorted –outSAMstrandField intronMotif –outFilterIntronMotifs RemoveNoncanonical –outReads UnmappedFastx”. For RNA-seq analyses, gene-level read quantification was derived using the subread-1.6.4 package (featureCounts) with a GTF annotation file (Gencode M22 or M25) and parameters “-s 2 -t exon -g gene_name”. For RIP-seq analyses, transcript-level reads were quantified with Salmon-0.13.1 using default parameters. Differential expression analysis was performed using DESeq2-1.22.1 with an adjusted *P* value threshold of 0.05 within R version 3.5.1 (https://www.R-project.org/). Gene Ontology term enrichment analysis for biological processes was performed using the Protein Analysis Through Evolutionary Relationships (PANTHER) classification system version 16.0 (http://www.pantherdb.org/) and a FDR *P* value threshold of 0.05 (Fisher’s exact test). Molecular interaction network was drawn using Cytoscape ver. 3.6.1 and an edge cutoff value of 0.375. Edinburgh Mouse Atlas Project Anatomy (EMAPA) term enrichment analysis was performed using the Mouse Genome Informatics (MGI; http://www.informatics.jax.org/) resource at The Jackson Laboratory and a *P* value threshold of 0.05 (Holm-Bonferroni method).

### Immunofluorescent labeling and microscopy

WT and *Fmr1*-KO mouse brains were harvested after transcardial perfusion with 4% paraformaldehyde in phosphate-buffered saline. Dissected brains were cryo-sectioned and immunofluorescently labeled as previously described [46], with the following modifications: 14 μm sections were boiled for 10 min in 10 mM Citrate Buffer, pH 6 (Sigma Aldrich). Following overnight incubation at 4 °C with primary antibodies mouse anti-FMRP (1:75, Developmental Studies Hybridoma Bank 7G1-1) and rabbit anti-p-UPF1 S1116 (1:1350; Millipore Sigma 07-1016), sections were washed in Tris-buffered saline (TBS) and subsequently labeled with Alexa Fluor 488-anti-rabbit IgG (1:1000; Thermo Fisher Scientific) or mouse-on-mouse kit (MOM, Vector Laboratories) with streptavidin-Cy3 (1:1000). After labeling with 1 μg/ml of 4′, 6-diamidine-2′-phenylindole dihydrochloride (DAPI, Sigma-Aldrich), sections were washed, and mounted with ProLong Gold Antifade Reagent (Thermo Fisher Scientific).

In vitro cultures of cortical cells isolated from P1 WT mice were fixed in 4% paraformaldehyde in PBS, permeabilized with 0.2% v/v Triton X-100 in PBS for 10 min, and blocked with 3% bovine serum albumin (Rockland) in TBS containing 0.1% v/v Tween 20 (TBST). After washing with TBST, coverslips were incubated with primary antibody overnight at 4 °C: mouse anti-FMRP (1:250, Millipore Sigma, MAB2160); rabbit anti-UPF1 (1:500) [43]; or rabbit anti-p-UPF1 S1116 (1:500; Millipore Sigma 07-1016). Coverslips were extensively washed with TBST and subsequently incubated with secondary antibodies Alexa Fluor 488-anti-mouse IgG (1:1000; Thermo Fisher Scientific) or Alexa Fluor 568-anti-rabbit IgG (1:1000; Thermo Fisher Scientific), followed by 1 μg/ml DAPI (Sigma-Aldrich) for 1 h at room temperature. After washing with TBST, coverslips were mounted using ProLong Gold Antifade Reagent. Immunofluorescent images were captured on a Leica TCS SP5 II laser scanning microscope, Nikon A1R HD confocal microscope, or Olympus FV-1000 confocal laser scanning microscope. Montages were assembled using Leica LAS AF or Fluoview 4.2, Image J (Fiji, Version 2.1.0) and Adobe Illustrator software. The quantitative analysis of protein colocalization was performed using Image J *coloc2* plugin software.

## Supplementary Information

**Additional file 1.** This file contains one supplementary table (Table S1).

**Additional file 2.** This file contains two supplementary figures (Figures S1-S2).

**Additional file 3.** This file contains one supplementary table (Table S2).

**Additional file 4.** This file contains one supplementary table (Table S3).

**Additional file 5.** Uncropped blots.

**Additional file 6.** Review history.

## Data Availability

RNA-seq and RIP-seq data are available in the Gene Expression Omnibus (GEO) under the accession number GSE180137 and NCBI Sequence Read Archive under the accession number PRJNA746942 (https://www.ncbi.nlm.nih.gov/geo/query/acc.cgi?acc=GSE180137 [47]). The data used for statistical analyses are provided in Additional file 4: Table S3.
