# Supplementary material for: NMD abnormalities during brain development in the Fmr1-knockout mouse model of fragile X syndrome
Source: Genome Biol. 2021 Nov 16;22:317. doi: 10.1186/s13059-021-02530-9 (PMC8597091; doi:10.1186/s13059-021-02530-9)
Supplement: Supplementary file 2 — Additional file 2. This file contains two supplementary figures (Figures S1-S2). [file 13059_2021_2530_MOESM2_ESM.pdf]

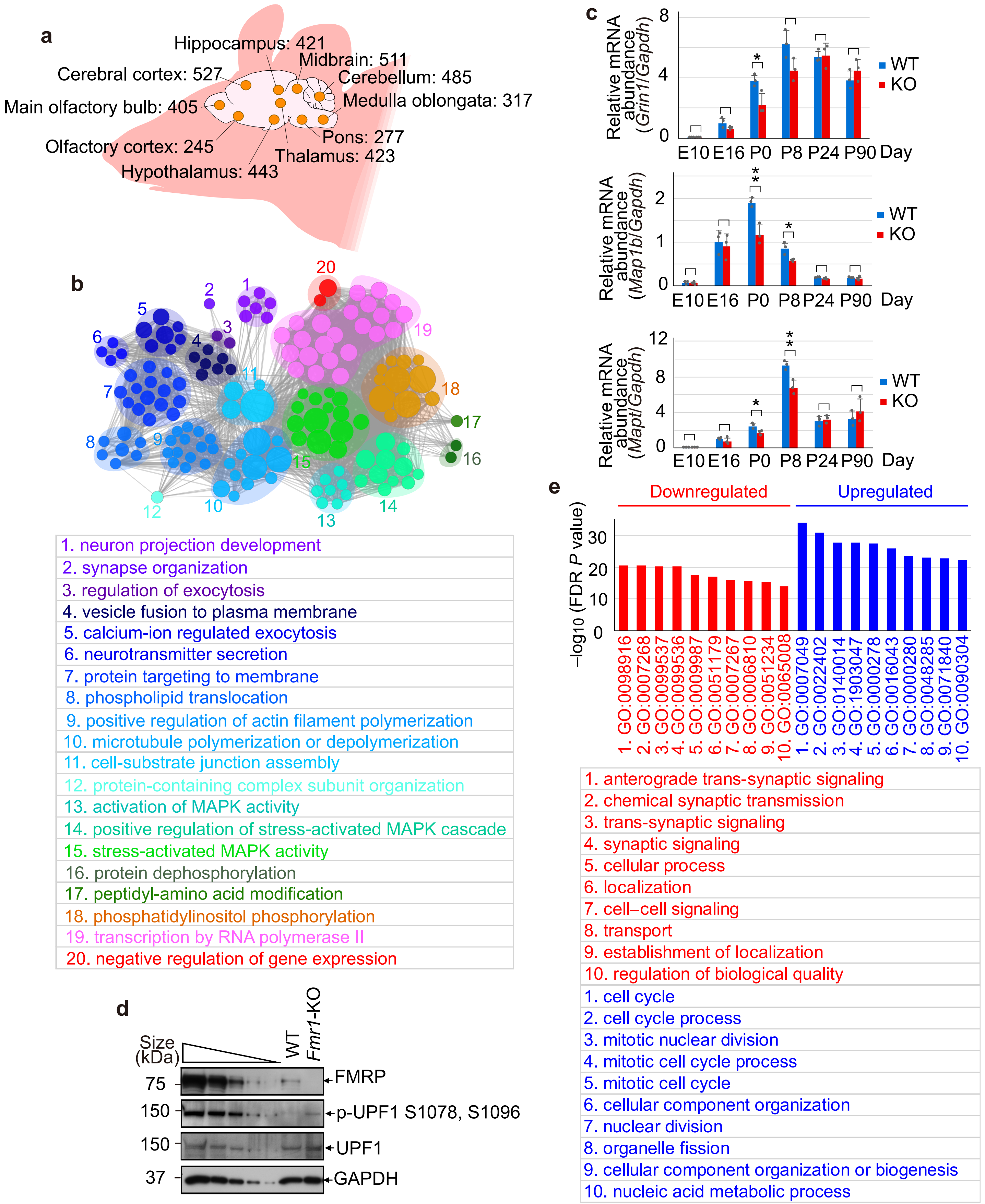

Fig. S1. Mouse NMD targets function in neuronal pathways. **a** EMAPA term enrichment analysis of the 1027 high-confidence NMD targets defined in Fig. 1a (false discovery rate [FDR] P-value < 0.05). The number of NMD targets identified for the specified brain region of adult mice is provided. **b** Network analysis using the GO term enrichment analysis dataset for NMD targets defined in Fig. 1a. **c** Histogram representation of RT-qPCR quantitations of the specified NMD targets, normalized to the level of *Gapdh* mRNA, using samples analyzed in Fig. 1d and e. The normalized levels in WT E16 mice are defined as 1. Means with S.D., where n = 3 (WT) and 3 (*Fmr1*-KO). (*)P < 0.05 or (**)P < 0.01 compares *Fmr1*-KO cells relative to WT cells (two-sided unpaired t-test). Note that it was not possible to reliably quantitate pre-mRNA levels from which each NMD target derives. However, one can compare the level of each NMD target, normalized to *Gapdh* mRNA, in WT and *Fmr1*-KO samples at each time point. **d** Western blot demonstrating that the level of p-UPF1 is elevated in 2-day cultures of P1 cortex from *Fmr1*-KO mice relative WT mice. **e** GO term enrichment analysis for either upregulated or downregulated transcripts in Fig. 1h. Only the top ten ranked functional terms are shown.

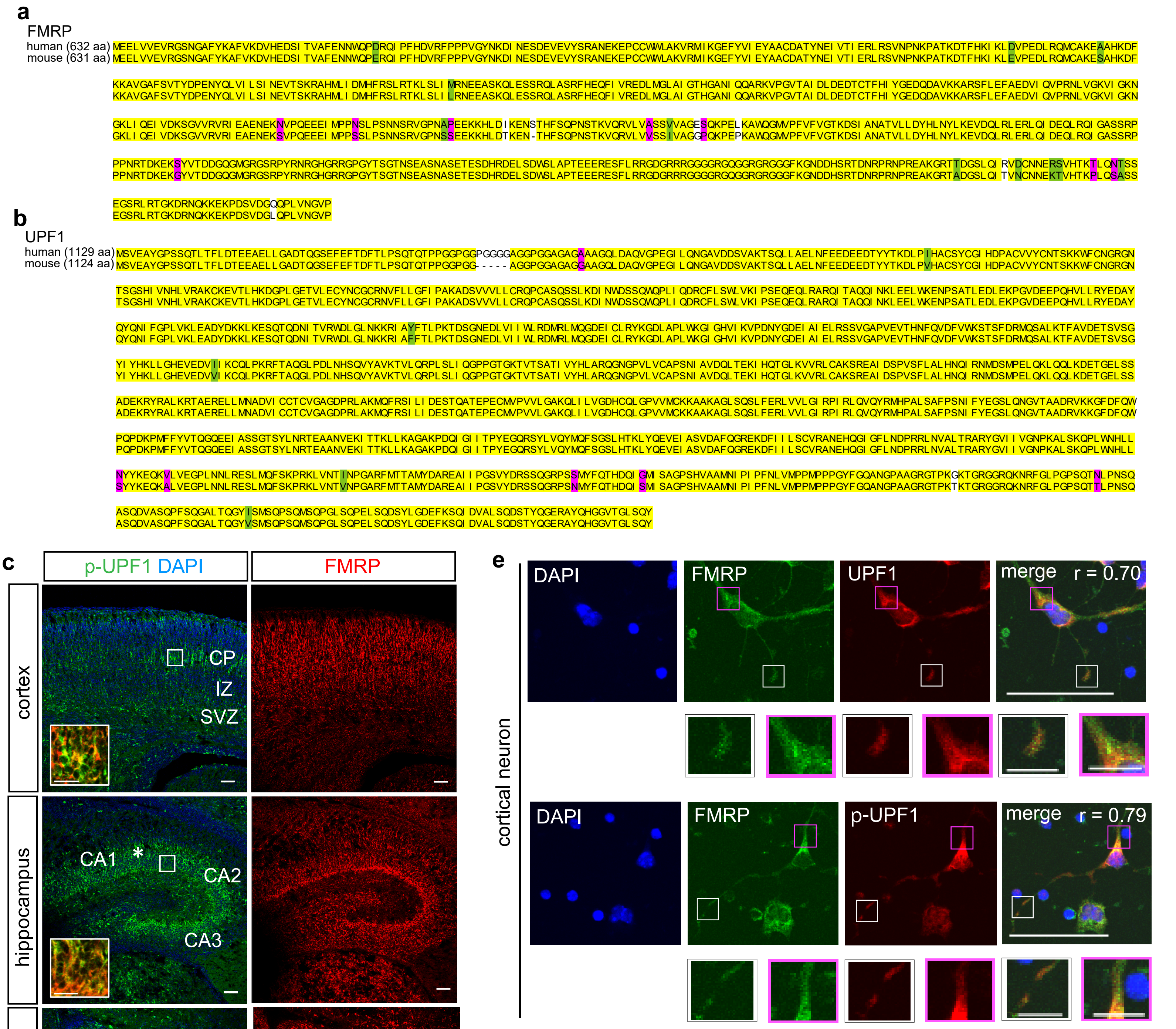

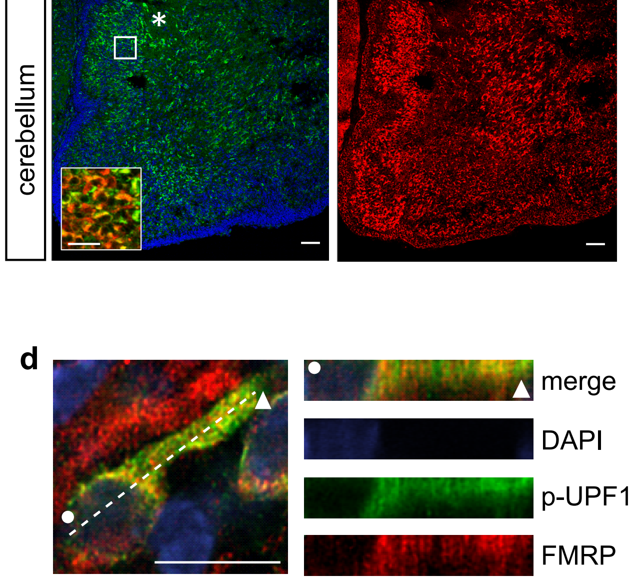

Fig. S2. Alignments of human and mouse FMRP and human and mouse UPF1; co-localized expression of p-UPF1 and FMRP in WT mouse P0 cortex, hippocampus and cerebellum. **a** Pairwise alignment of human and mouse FMRP (ENST00000370475.9 and ENSMUST00000114655.8, respectively). Yellow, green and pink highlights denote amino acids that are, respectively, perfectly identical, strongly similar, or weakly similar based on the PAM250 matrix. **b** As in a, but for human and mouse UPF1 (ENST00000599848.5 and ENSMUST00000075666.8, respectively). **c** Confocal image of p-UPF1 (green, left) and FMRP (red, right) immunostaining of cortex, hippocampus, and cerebellum from P0 WT mice. Nuclei have been stained using DAPI (blue). CP, cortical plate; IZ, intermediate zone; SVZ, subventricular zone; CA, Cornus Ammon region. The asterisk (*) denotes molecular layer. Scale bar, 50 µm; Insets provide magnified views of the specified boxed regions of interest. Scale bar, 20 µm. **d** Magnified confocal image of cortex at P0 (left). A line is drawn in the merged image through a single cell showing DAPI (blue), p-UPF1 (green) and FMRP (red) staining. Z-stack projections are shown for 0.125 µm slices (right). Scale bar, 10 µm. **e** Confocal images of neurons from 2-day cultures of WT P1 cortex using the specified antibody or DAPI to stain nuclei. r, Pearson's correlation coefficient. Scale bar, 50 µm. Insets provide magnified views of the specified boxed regions of interest. Scale bar, 10 µm.
