# Supplementary material for: NMD abnormalities during brain development in the Fmr1-knockout mouse model of fragile X syndrome
Source: Genome Biol. 2021 Nov 16;22:317. doi: 10.1186/s13059-021-02530-9 (PMC8597091; doi:10.1186/s13059-021-02530-9)
Supplement: Supplementary file 3 — Additional file 3. This file contains one supplementary table (Table S2). [file 13059_2021_2530_MOESM3_ESM.docx]

**Table S2: Oligonucleotides used in this study**

| **Oligonucleotides** | **Company** | **Catalog #** |
| --- | --- | --- |
| Random Hexamers (50 μM) | Thermo Fisher Scientific | N8080127 |
| Silencer Negative Control #1 siRNA | Thermo Fisher Scientific/Ambion | AM4636 |
| *Upf1* siRNA: 5'- UCAAGGUUCCUGAUAAUUA-3' | Dharmacon/GE Healthcare | N.A. |
| *Grin1* S: 5'- AGAGCCCGACCCTAAAAAGAA-3' | Integrated DNA Technologies | N.A. |
| *Grin1* AS: 5'- CCCTCCTCCCTCTCAATAGC-3' | Integrated DNA Technologies | N.A. |
| *Grin1* pre-S: 5'- CCGCCACCCTTAGTTGTCTA-3' | Integrated DNA Technologies | N.A. |
| *Grin1* pre-AS: 5'- TTCCCCTTACCGTGTCTTTG-3' | Integrated DNA Technologies | N.A. |
| *Map1b* S: 5'- AAAGACCACCACCACTCCTG-3' | Integrated DNA Technologies | N.A. |
| *Map1b* AS: 5'- TGTTGCTGTGGTTGGGAATA-3' | Integrated DNA Technologies | N.A. |
| *Map1b* pre-S: 5'- AAAGACCACCACCACTCCTG-3' | Integrated DNA Technologies | N.A. |
| *Map1b* pre-AS: 5'- TCCACCATTTCTGGTCTTCC-3' | Integrated DNA Technologies | N.A. |
| *Mapt* S: 5'- GAATGTCAGGTCGAAGATTGGC-3' | Integrated DNA Technologies | N.A. |
| *Mapt* AS: 5'- TGGACTGGACGTTGCTAAGAT-3' | Integrated DNA Technologies | N.A. |
| *Mapt* pre-S: 5'- CTCCCCCTAAGTCACCATCA-3' | Integrated DNA Technologies | N.A. |
| *Mapt* pre-AS: 5'- GGAGCAAGTCTGGCTAGGTG-3' | Integrated DNA Technologies | N.A. |
| *Gabbr1* S: 5'-CTCCTGGACGGATATGGACAC-3' | Integrated DNA Technologies | N.A. |
| *Gabbr1* mRNA R: 5'- GTCGGGGTCACATCGGAAAT-3' | Integrated DNA Technologies | N.A. |
| *Gabbr1* pre-S: 5'- ATCAGGTACCGTGGCTTGAC-3' | Integrated DNA Technologies | N.A. |
| *Gabbr1* pre-AS: 5'- TGGTCTGCAACTGAGAATGG-3' | Integrated DNA Technologies | N.A. |
| *Olfm1* S: 5'-GCCATAAGCAGCATCTAGCC-3' | Integrated DNA Technologies | N.A. |
| *Olfm1 AS*: 5'-ATAGGCGCCAATCTCCTCTT-3' | Integrated DNA Technologies | N.A. |
| *Olfm1* pre-S: 5'-AGCTGAGGCAGCTACTGGAG-3' | Integrated DNA Technologies | N.A. |
| *Olfm1* pre-AS: 5'- TCCTAGCCACTCGGTCATCT-3' | Integrated DNA Technologies | N.A. |
| *Sema6d* S: 5'- AATATCCGGTTTTTAGAGGACGC-3' | Integrated DNA Technologies | N.A. |
| *Sema6d* AS: 5'-TGCTTGCCTTTCATAGCACAAT-3' | Integrated DNA Technologies | N.A. |
| *Sema6d* pre-S: 5'- GGACTGCCACGgtaagacag-3' | Integrated DNA Technologies | N.A. |
| *Sema6d* pre-AS: 5'- CAAATGCGGTCCAAAGGAGT-3' | Integrated DNA Technologies | N.A. |
| *Sez6* S: 5'- GCCCCTGTAGCTGGAATTTCT-3' | Integrated DNA Technologies | N.A. |
| *Sez6* AS: 5'- GCAGGAACGACTGGTTAGCC-3' | Integrated DNA Technologies | N.A. |
| *Sez6* pre-S: 5'- TTGGCTAACCAGTCGTTCCT-3' | Integrated DNA Technologies | N.A. |
| *Sez6* pre-AS: 5'- CCTTCAGCTCCTCACTGACC-3' | Integrated DNA Technologies | N.A. |
| *Gria2* S: 5'- TTCTCCTGTTTTATGGGGACTGA-3' | Integrated DNA Technologies | N.A. |
| *Gria2* AS: 5'- CTACCCGAAATGCACTGTATTCT-3' | Integrated DNA Technologies | N.A. |
| *Gria2* pre-S: 5'- ATTTCGGGTAGGGATGGTTC-3' | Integrated DNA Technologies | N.A. |
| *Gria2* pre-AS: 5'-accatccttcactggcattc-3' | Integrated DNA Technologies | N.A. |
| *Elavl4* S: 5'-CTCCAGACCAAAACCATAAAGGT-3' | Integrated DNA Technologies | N.A. |
| *Elavl4* AS: 5'-TGATGCGACCGTATTGAGAGAA-3' | Integrated DNA Technologies | N.A. |
| *Elavl4* pre-S: 5'- CCCAGTCTCTCTGCTCCATC-3' | Integrated DNA Technologies | N.A. |
| *Elavl4* pre-AS: 5'- TTGTATTGGATGTCGGTCCA-3' | Integrated DNA Technologies | N.A. |
| *Myt1* S: 5'-ATGCCTCTCACAGGAGTGCT-3' | Integrated DNA Technologies | N.A. |
| *Myt1* AS: 5'- TTTCCAGCAAAGGTTGCTCT-3' | Integrated DNA Technologies | N.A. |
| *Myt1* pre-S: 5'- ATGCCTCTCACAGGAGTGCT-3' | Integrated DNA Technologies | N.A. |
| *Myt1* pre-AS: 5'- CCCTGATTGTTGTTGACGTG-3' | Integrated DNA Technologies | N.A. |
| *E2f7* S: 5'- AGGATGCGTTCGTGAACTCC-3' | Integrated DNA Technologies | N.A. |
| *E2f7* AS: 5'- TGACAAGGGGTAGCTCGGATA-3' | Integrated DNA Technologies | N.A. |
| *E2f7* pre-S: 5'- CGCCAGCCCAGACATAAGAG-3' | Integrated DNA Technologies | N.A. |
| *E2f7* pre-AS: 5'- GGGGAAGTTTTACAAAGCCCTC-3' | Integrated DNA Technologies | N.A. |
| *Gapdh* S: 5'- AACTTTGGCATTGTGGAAGG-3' | Integrated DNA Technologies | N.A. |
| *Gapdh* AS: 5'- ACACATTGGGGGTAGGAACA-3' | Integrated DNA Technologies | N.A. |
| *Gapdh* pre-S: 5'- CTCAGCTCCCCTGTTTCTTG-3' | Integrated DNA Technologies | N.A. |
| *Gapdh* pre-AS: 5'- GAATTTGCCGTGAGTGGAGT-3' | Integrated DNA Technologies | N.A. |
