# Supplementary figures and images for: NMD abnormalities during brain development in the Fmr1-knockout mouse model of fragile X syndrome
Source: Genome Biol. 2021 Nov 16;22:317. doi: 10.1186/s13059-021-02530-9 (PMC8597091; doi:10.1186/s13059-021-02530-9)

Figure 1b

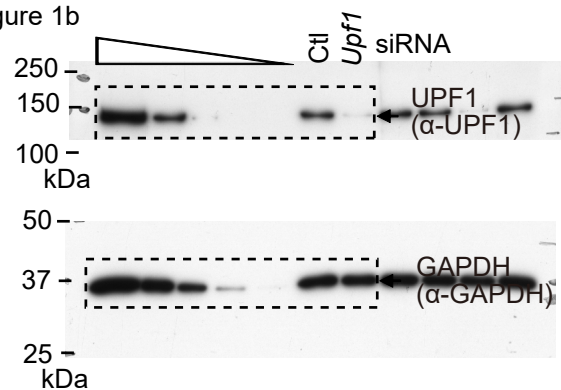

Figure 1c

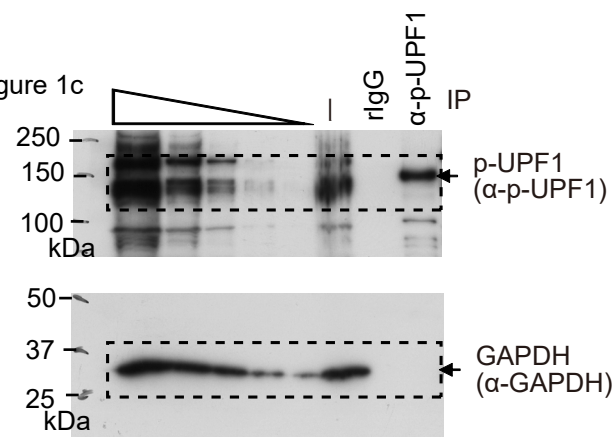

Figure 1d

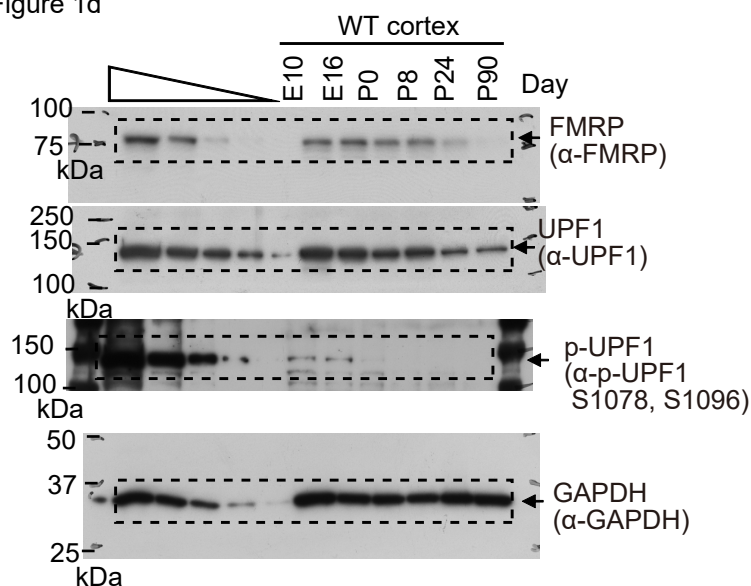

Figure 1e

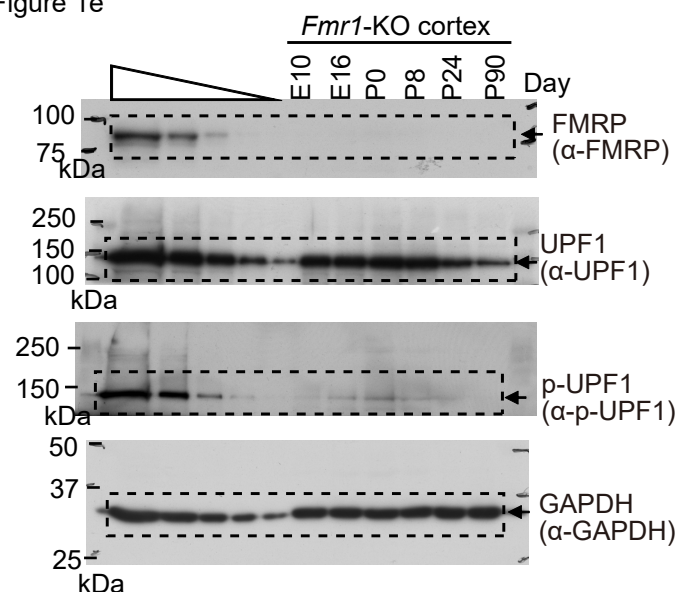

Figure 2a

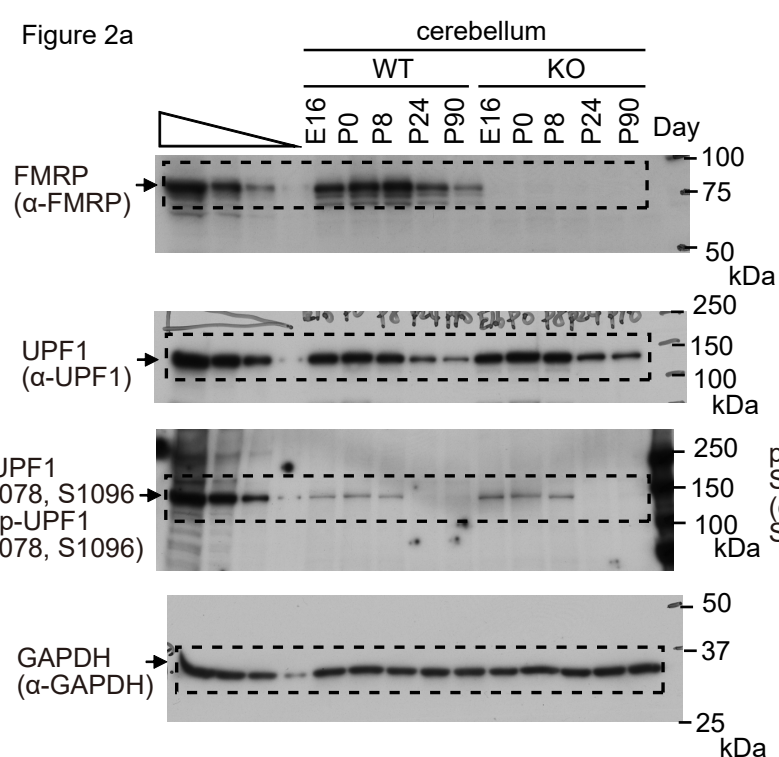

Figure 2d

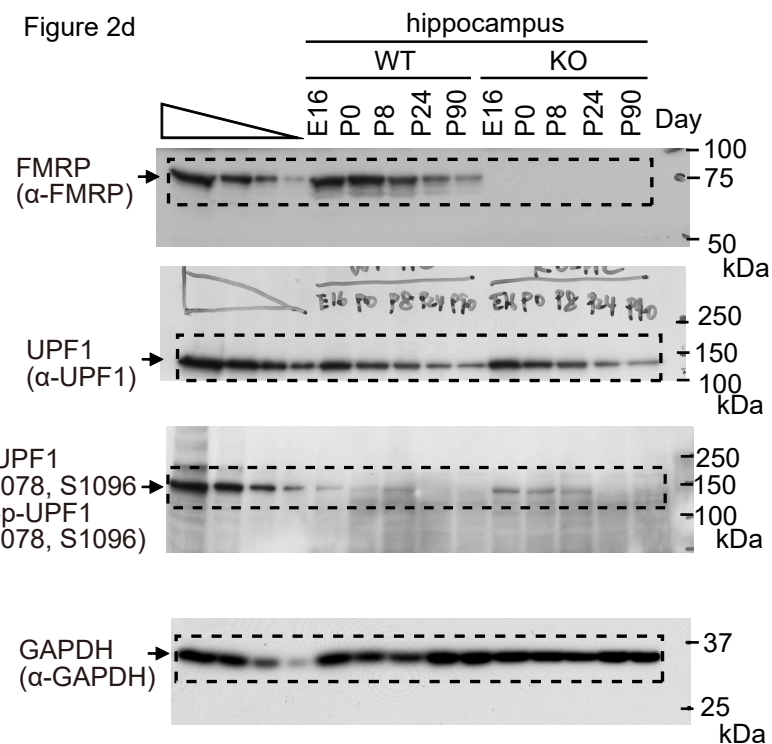

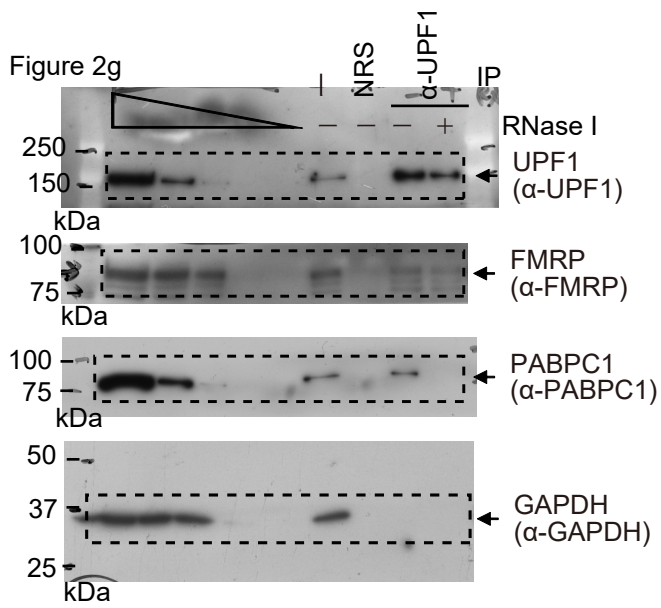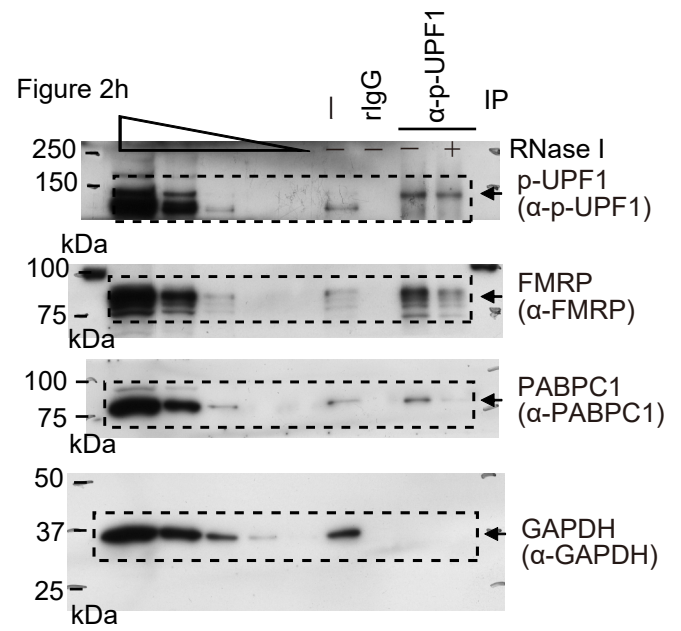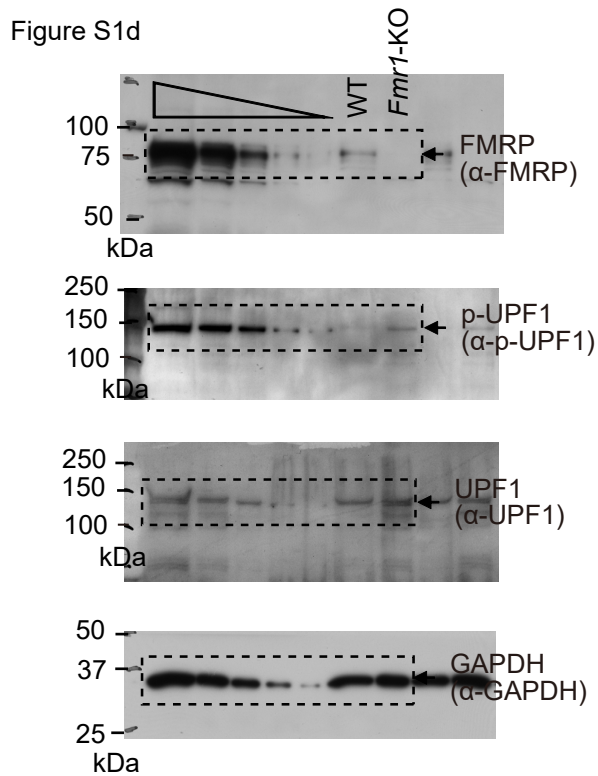

Supplement: Supplementary file 5 — Additional file 5. Uncropped blots. [file 13059_2021_2530_MOESM5_ESM.pdf]
