# Supplementary material for: NMD abnormalities during brain development in the Fmr1-knockout mouse model of fragile X syndrome
Source: Genome Biol. 2021 Nov 16;22:317. doi: 10.1186/s13059-021-02530-9 (PMC8597091; doi:10.1186/s13059-021-02530-9)
Supplement: Supplementary file 6 — Additional file 6. Review history. [file 13059_2021_2530_MOESM6_ESM.docx]

Review History

**First round of review**

**Reviewer 1**

**Are you able to assess all statistics in the manuscript, including the appropriateness of statistical tests used?**

Yes, and I have assessed the statistics in my report.

**Comments to author:**

This study identified NMD targets in a mouse cell line and characterized the developmental profile of NMD, using UPF1 as an indicator, in wildtype and Fmr1 knockout (KO) mice. This is an important study with several data sets that are prerequisite to the use of mouse models for mechanistic studies of NMD contribution to FXS pathology. The manuscript is well written with sound experimental design, sufficient controls, and appropriate statistical analyses. I only have several suggestive comments for further clarifications.

1. The authors provided the rationale of identifying mouse NMD targets using N2A cells, which is reasonable, and used the resultant target list for analyzing cortical neurons. A statement that this approach may overlook cell-type specific targets would help reduce confusion. Additionally, a comparison of NMD targets identified in N2A cells (this study) to UPF1-bound RNAs in mouse embryonic stem cells (Hurt et al., 2013) would be informative to the field and may further support the relevance of applying NMD targets of N2A cells to cortical neurons.

2. It is not clear why the P1 cortex was cultured for 2 days before used for RNA-seq analysis (Fig. 1h).

3. The localization analyses (Fig. S2) showed the immuno-detection of both FMRP and UPF1 in the cell body and neurite of developing neurons. High-magnification images are expected to argue a more likely true co-localization vs. co-exist in cell body (or neurites) independently.

4. One novel observation of this study is the timing of NMD alterations in Fmr1 KO mouse brain regions. It would be informative to discuss possible factors that may define this timing. Does FMRP binds to UPF1 in co-IP or co-localize with UPF1 immunoreactivity at P90 when NMD is comparable between WT and KO mice? If not, this could provide a possible mechanism.

5. The authors interpreted the results by considering FMRP mainly as a translational repressor. Given that FMRP may regulate RNA synthesis and stability via other mechanisms such as interactions with microRNAs, a discussion with this regard may further strengthen the manuscript.

Others:

Line 321: delete "a" before "secondary antibodies"

**Reviewer 2**

**Are you able to assess all statistics in the manuscript, including the appropriateness of statistical tests used?**

Yes, and I have assessed the statistics in my report.

**Comments to author:**

This short report by Kurosaki, Maquat, et al., investigates the role of Fmr1 in regulating nonsense-mediated mRNA decay during mouse brain development. The Maquat lab previously showed physical and functional interactions between FMRP and NMD in several human cell types, including neurons differentiated in vitro. Here, they extend their previous finding that loss of Fmr1 downregulates at least some NMD target mRNAs to cultured cortical neurons and primary tissues from brains of wild-type and Fmr1 knockout mice. Using RIP-Seq and RNA-seq, the authors identify a population of direct NMD targets in mouse N2A cells, which they show are on average modestly downregulated in neurons cultured from Fmr1 knockout mouse brains. They further show that Upf1 phosphorylation timing or levels are altered in the cortex, cerebellum, and hippocampus of Fmr1 knockout mice and that mouse Fmr1 and Upf1, like their human homologs, interact in an RNase-insensitive manner. The role of Fmr1 in regulating NMD is an important and interesting topic, and confirmation that several observations from the authors' interesting prior study hold in mouse cerebellar and hippocampal tissues and/or cultured neurons is welcome. However, this manuscript would be significantly strengthened by direct analysis of RNA levels in brain tissues, rather than the current use of p-Upf1 levels as a proxy for NMD activity.

Major points:

1. Most of the figures replicate experiments that they previously performed in one or more human cell types, including neurons derived from iPSCs. A more in-depth examination of changes in NMD efficiency during brain development using RNA-seq rather than Upf1 phosphorylation would be one way to add new insight into the physiological function of Fmr1 in neuronal NMD.

2. A major issue with the manuscript is the use of Upf1 phosphorylation as a proxy for NMD efficiency. Upf1 phosphorylation is required for NMD, but that does not always mean that cells with more highly phosphorylated Upf1 conduct NMD more efficiently. Others have reported the opposite phenomenon, most notably Durand et al., NSMB 2016, who showed that cells depleted of various decay factors have higher levels of Upf1 phosphorylation. These observations are consistent with findings from several groups that depletion of SMG5/7 causes increased Upf1 phosphorylation.

3. The authors provide titrations on Western blots, but for the p-Upf1 detection, most of the bands have intensities at or below the bottom of the titration range. Given the importance of the Western quantification to the paper, more convincing evidence that the quantified signals are in the linear range of the assay is needed.

4. Do the cultured neurons used for the experiments in Figure 1i-j have elevated p-Upf1?

Minor points:

1. Line 48--quantification of the mean enrichment would be more informative than the p value (since no info is given regarding the tests used, sample sizes, or comparisons made).

2. Figure S1: I'm not sure how to evaluate the statistic that > 50% of the NMD targets are expressed in cerebral cortex and ~40% in the hippocampus . Are these tissue-specific mRNAs or broadly expressed?

3. Figure S1: The results of GO analyses are strongly dependent on the background set of genes used to evaluate the null hypothesis, so this information should be included in the methods.

4. Abstract: The basis for the claim that the "findings identify the importance of NMD-encoded proteins to brain development" is not clear from the data shown. The previous study used NMD inhibition to rescue neuronal differentiation, but no functional experiments establishing the role of NMD in mouse brain development are included here.

**Authors Response**

**Point-by-point responses to the reviewers’ comments:**

Reviewer #1: This study identified NMD targets in a mouse cell line and characterized the developmental profile of NMD, using UPF1 as an indicator, in wildtype and Fmr1 knockout (KO) mice. This is an important study with several data sets that are prerequisite to the use of mouse models for mechanistic studies of NMD contribution to FXS pathology. The manuscript is well written with sound experimental design, sufficient controls, and appropriate statistical analyses. I only have several suggestive comments for further clarifications.

*Our response: We are grateful for the appreciation of our research – our new foray into using the Fmr1-KO mouse to lay the ground work for future developmental, mechanistic and therapeutic studies.*

1. The authors provided the rationale of identifying mouse NMD targets using N2A cells, which is reasonable, and used the resultant target list for analyzing cortical neurons. A statement that this approach may overlook cell-type specific targets would help reduce confusion. Additionally, a comparison of NMD targets identified in N2A cells (this study) to UPF1-bound RNAs in mouse embryonic stem cells (Hurt et al., 2013) would be informative to the field and may further support the relevance of applying NMD targets of N2A cells to cortical neurons.

*Our response: Hurt et al., defined NMD targets using two methods: (1) mRNA upregulation upon UPF1 downregulation or upon inhibition of translation by cycloheximide treatment, and (2) anti-UPF1 CLIP, the latter of which is not ideal since we have shown that UPF1 binds nonspecifically to any physically accessible RNA (Kurosaki et al., 2014). Such nonspecific binding confounds the Hurt et al. data by contributing to background binding. We have found that using differences in UPF1 binding is reliable only when comparing a premature termination codon (PTC)-containing mRNA to its PTC-free counterpart, so as to compare differences in 3′UTR length (Kurosaki and Maquat, 2013, Proc. Nat. Acad. Sci.): it is not reliable as a means to identify NMD targets transcriptome-wide. Nevertheless, this Reviewer is asking for a comparison of NMD targets that we have defined (as upregulated upon UPF1 downregulation and also bound by p-UPF1) using N2A cells to UPF1-bound mRNAs defined by Hurt and colleagues. There were 226 UPF1-bound mRNAs that were defined as high-confidence by Hurt et al., of which they found only 38 that were upregulated by UPF1 siRNA and cycloheximide treatment. Comparing our analyses with theirs, we defined 36 NMD targets among their 226, and we defined 6 NMD targets among their 38. Considering these comparisons, and for reasons stated above, we do not believe that comparing our data to the UPF1-binding data of Hurt et al. would provide additional insights.*

*In addressing this Reviewer’s comments, we now state the following on page 5, lines 117-120. “However, it should be noted that utilizing NMD targets that we established for N2A neuroblastoma cells to define those expressed in 2-day cultures of P1 cortex would overlook NMD targets expressed in the 2-day cultures but not in N2A cells.”*

2. It is not clear why the P1 cortex was cultured for 2 days before used for RNA-seq analysis (Fig. 1h).

*Our response: By isolating and culturing cortical neurons, we generate a much more uniform cell-type that largely consists of cortical neurons. For example, the isolation protocol results in removal of blood vessels using cell strainers, and immune-depletion of ACSA-2-positive astrocytes and myelin using anti-ACSA-2 and anti-myelin microbeads. Selective growth conditions in Neurobasal medium on a poly-L-lysine/laminin substrate further preferentially support neuron survival. Generating a more uniform cell-type is important because the mRNA production depends on expression of the genes from which they derive and, thus, will vary among cell-types (see, e.g., Donnard et al., Single cell transcriptomics reveals dysregulated cellular and molecular networks in a fragile X syndrome model, bioRxiv 2020). We now offer an explanation as follows on page 5, lines 113-114. “Our rationale for performing RNA-seq using P1 cortex cultured for two days in vitro rather than using cortex per se was to reduce the cell-type heterogeneity that typifies cortex.”*

3. The localization analyses (Fig. S2) showed the immuno-detection of both FMRP and UPF1 in the cell body and neurite of developing neurons. High-magnification images are expected to argue a more likely true co-localization vs. co-exist in cell body (or neurites) independently.

*Our response: We have now included a new Fig. S2d, which shows a magnified confocal image of P0 cortex (left) in which a line is drawn in the merged image through a single cell showing DAPI (blue), p-UPF1 (green) and FMRP (red) staining. Also shown are Z-stack projections of 0.125µm slices (right).*

*Additionally, in Fig. S2e, we have supplemented the confocal images of neurons from 2-day cultures of WT P1 cortex (Scale bar, 50 μm) with insets that provide magnified views of the specified boxed regions of interest (Scale bar, 10 µm).*

*On page 7, lines 162-167, these new figures are referenced as follows. “At the subcellular level, FMRP, UPF1 and p-UPF1 were co-localized in both the soma and the neurites of cortical neurons in vivo and in vitro (Additional file 4: Fig. S2d and e, respectively) by confocal imaging. Since the co-localization of FMRP and p-UPF1 is known to also occur on translating NMD targets in human cells [6], our localization of both proteins to neurites (Additional file 4: S2d and e) is consistent with the recent report that FMRP promotes RNA localization to neuronal projections in mouse neuronal catecholaminergic CAD cells [23].”*

4. One novel observation of this study is the timing of NMD alterations in Fmr1 KO mouse brain regions. It would be informative to discuss possible factors that may define this timing. Does FMRP binds to UPF1 in co-IP or co-localize with UPF1 immunoreactivity at P90 when NMD is comparable between WT and KO mice? If not, this could provide a possible mechanism.

*Our response: Of course, we don’t know for sure about mechanism, but this Reviewer raises an important question about differences in the timing of NMD alterations between WT and Fmr1-KO mouse brain regions. By P90, p-UPF1 levels are comparable between WT and Fmr1-KO mice for each of the three tissues examined and also the lowest of all timepoints assayed. While the variable is the presence and absence of FMRP, we do not yet understand mechanism other than, as stated, the absence of FMRP hyperactivates NMD.*

*We can enrich our statements, however, and do this after the statement “NMD is most active at E10 and E16 in WT mice, during which time p-UPF1 levels are just starting to increase in Fmr1-KO mice. NMD efficiency then precipitously drops by P0 in WT mice but remains abnormally high through P24 in Fmr1-KO mice (Fig. 1f and g).” After this statement, we now add the following to page 4, on lines 101-105. “Given these findings, and that NMD has been shown to promote the stem-cell state of mouse neuronal stem cells derived from E14.5 brains grown as neurospheres and to decrease in efficiency once these cells were differentiated by withdrawing growth factors [16], it follows that neuronal cell differentiation would be expected to be abnormally delayed in Fmr1-KO mice.”*

5. The authors interpreted the results by considering FMRP mainly as a translational repressor. Given that FMRP may regulate RNA synthesis and stability via other mechanisms such as interactions with microRNAs, a discussion with this regard may further strengthen the manuscript.

*Our response: We agree and have added the following to the Discussion on page 9, lines 198-201 “To further complicate mechanistic differences between WT and Fmr1-KO mice, FMRP has been shown to regulate many aspects of gene expression beyond mRNA translation, including chromatin modification, RNA synthesis and processing, adenosine deaminases acting on RNA, and microRNA associations with RNA-induced silencing complexes [33].”*

Others:

Line 321: delete "a" before "secondary antibodies"

*Our response: Done, thank you.*

Reviewer #2: This short report by Kurosaki, Maquat, et al., investigates the role of Fmr1 in regulating nonsense-mediated mRNA decay during mouse brain development. The Maquat lab previously showed physical and functional interactions between FMRP and NMD in several human cell types, including neurons differentiated in vitro. Here, they extend their previous finding that loss of Fmr1 downregulates at least some NMD target mRNAs to cultured cortical neurons and primary tissues from brains of wild-type and Fmr1 knockout mice. Using RIP-Seq and RNA-seq, the authors identify a population of direct NMD targets in mouse N2A cells, which they show are on average modestly downregulated in neurons cultured from Fmr1 knockout mouse brains. They further show that Upf1 phosphorylation timing or levels are altered in the cortex, cerebellum, and hippocampus of Fmr1 knockout mice and that mouse Fmr1 and Upf1, like their human homologs, interact in an RNase-insensitive manner. The role of Fmr1 in regulating NMD is an important and interesting topic, and confirmation that several observations from the authors' interesting prior study hold in mouse cerebellar and hippocampal tissues and/or cultured neurons is welcome. However, this manuscript would be significantly strengthened by direct analysis of RNA levels in brain tissues, rather than the current use of p-Upf1 levels as a proxy for NMD activity.

*Our response: We are grateful for this Reviewer’s positive comments. We now include RT-qPCR to quantitate defined NMD targets (Grin1, Map1b and Mapt mRNAs) normalized to Gapdh mRNA to corroborate that levels of p-UPF1 provide a reliable proxy for NMD efficiency. These new data are shown in new Fig. S1c for cortex during development. Please note that instead of performing RNA-seq on, e.g., whole cortex, we instead chose to perform what we believe is a much more useful analysis of NMD targets in WT and Fmr1-KO mice by culturing P1 cortex for 2 days. By culturing, we generate a much more uniform cell-type that largely consists of cortical neurons. For example, the isolation protocol results in the removal of blood vessels and a depletion of ACSA1-positive astrocytes. Selective growth conditions in Neurobasal medium on a poly-L-lysine substrate further preferentially support neuron survival. Generating a more uniform cell-type is important because the production of NMD targets depends on expression of the genes from which the targets derive and, thus, will vary among cell-types. Given the current push toward single-cell sequencing, we believe sequencing whole cortical, cerebellar or hippocampal tissues at this point would be much less valuable to readers/researchers than our RNA-seq analysis of cultured P1 cortex coupled with our defining NMD targets for N2A cells. Given the groundwork that we provide, we are hopeful that genomics-centric researchers will undertake single-cell sequencing, especially as the technology becomes more sensitive and mRNA levels can be normalized to pre-mRNA levels as a means to control for changes in gene transcription.*

Major points:

1. Most of the figures replicate experiments that they previously performed in one or more human cell types, including neurons derived from iPSCs. A more in-depth examination of changes in NMD efficiency during brain development using RNA-seq rather than Upf1 phosphorylation would be one way to add new insight into the physiological function of Fmr1 in neuronal NMD.

*Our response: Please see our response to the issue raised above. We agree that RNA-seq determinations of NMD efficiency during brain development would be useful. However, for these additional data, to be meaningful, they should derive from single-cell RNA-seq experiments. While we certainly can sequence RNA from the cortex, cerebellum and hippocampus at different times of development, comparing WT and Fmr1-KO mice, we do not believe that this very labor-intensive undertaking is of real value and is limited by the sequencing depth that can be achieved in single cells. Instead, what we have done is to use RNA deriving from samples analyzed in Fig. 1d and e and include RT-qPCR analysis of whole cortex as a function of developmental time. Our results corroborate our use of p-UPF1 as a proxy for NMD efficiency: for each time point, the level of each of three NMD targets changes in Fmr1-KO relative to WT samples as predicted from changes in the levels of p-UPF1. These results are included as new Fig. S1c. We now state the following on page 5, lines 106-109: “Consistent with FMRP loss hyperactivating NMD during development, RT-qPCR demonstrated that the abundance of each of three known NMD targets (Grin1, Map1b, and Mapt) in Fmr1-KO relative to WT mice were abnormally low in P0 and P8 cortex (Additional file 2: Fig. S1c).”*

*For additional justification for using p-UPF1 as a proxy for NMD efficiency, please see our discussion provided below. We have studied the dynamics of wild-type and mutated UPF1 and the consequences of UPF1 phosphorylation to NMD for decades.*

2. A major issue with the manuscript is the use of Upf1 phosphorylation as a proxy for NMD efficiency. Upf1 phosphorylation is required for NMD, but that does not always mean that cells with more highly phosphorylated Upf1 conduct NMD more efficiently. Others have reported the opposite phenomenon, most notably Durand et al., NSMB 2016, who showed that cells depleted of various decay factors have higher levels of Upf1 phosphorylation. These observations are consistent with findings from several groups that depletion of SMG5/7 causes increased Upf1 phosphorylation.

*Our response: This Reviewer is correct when noting that cells with an elevated level of p-UPF1 relative to cells that do not have an elevated level of p-UPF1 do not necessarily support an elevated efficiency of NMD. This conclusion derives from Durand et al., 2016 and, we wish to point out, our work that preceded the Durand paper: Kurosaki et al., 2014, Genes Dev. We entitled our paper “A post-translational regulatory switch on UPF1 controls targeted mRNA degradation”. The “switch”, which is an on-off switch, is phosphorylation-dephosphorylation.*

*Both papers note that it is the cycle of UPF1 phosphorylation and dephosphorylation that is required for efficient NMD. For example, we showed that UPF1 that is “stuck” in a hyperphosphorylated state, either because cells express a mutated UPF1 or express SMG5 siRNA or SMG7 siRNA, manifest inefficient NMD. Nevertheless, we and others have solid documentation that p-UPF1 serves as a reliable proxy for NMD activity unless there is an experimental manipulation of UPF1 catalytic activity or a disrupt SMG5-, SMG6- or SMG7- mediated functions initiated by their binding to p-UPF1: see not only Kurosaki et al., Genes Dev 2014; Durand et al., 2016; but also the following:*

*Imamachi, N., Salam, K. A., Suzuki, Y. & Akimitsu, N. A GC-rich sequence feature in the 3′ UTR directs UPF1-dependent mRNA decay in mammalian cells. Genome Res. 27, 407–418 (2017).*

*Isken, O. et al. Upf1 phosphorylation triggers translational repression during nonsense-mediated mRNA decay. Cell 133, 314–327 (2008).*

*Kashima, I. et al. Binding of a novel SMG-1–Upf1–eRF1–eRF3 complex (SURF) to the exon junction complex triggers Upf1 phosphorylation and nonsense-mediated mRNA decay. Genes Dev. 20, 355–367 (2006).*

*Lee, S. R., Pratt, G. A., Martinez, F. J., Yeo, G. W. & Lykke-Andersen, J. Target discrimination in nonsense-mediated mRNA decay requires Upf1 ATPase activity. Mol. Cell 59, 413–425 (2015).*

*Kurosaki, T., Miyoshi, K., Myers, J. R. & Maquat, L. E. NMD-degradome sequencing reveals ribosome-bound intermediates with 3′-end non-templated nucleotides. Nat. Struct. Mol. Biol. 25, 940–950 (2018).*

*Kurosaki T, Imamachi N, Pröschel C, Mitsutomi S, Nagao R, Akimitsu N, Maquat LE. Loss of the fragile X syndrome protein FMRP results in misregulation of nonsense-mediated mRNA decay. Nat Cell Biol. 23, 40-48 (2021).*

*Ohnishi, T. et al. Phosphorylation of hUPF1 induces formation of mRNA surveillance complexes containing hSMG-5 and hSMG-7. Mol. Cell 12, 1187–1200 (2003).*

*We are confident that our analyses of p-UPF1 levels in the paper under review do represent a reliable comparison of NMD efficiency in WT vs. Fmr1-KO cells.*

*All that noted, we undertook two additional analyses:*

*1. We now include as new Fig. S1c, RT-qPCR to quantitate the level of three NMD targets, normalized to the level of Gapdh mRNA. Please see our response to comment #1. These data corroborate that western blot quantitations of p-UPF1 levels do provide a reliable proxy for NMD efficiencies.*

*2. We now include as new Fig. S1d western blots demonstrating that the level of p-UPF1 is increased in P1 cortical neurons deriving from Fmr1-KO mice relative to WT mice after culturing for two days in vitro.*

*Since the Reviewer makes a point that other readers might raise, we have added the Durand et al, 2016 reference as follows to page 4, lines 95-97. “While the level of UPF1 in WT mice relative to Fmr1-KO mice remained comparable at all developmental stages that were assayed (Fig. 1d and e), the level of phosphorylated UPF1 (p-UPF1), which can be used as a proxy for NMD efficiency [6, 7, 15]…”.*

3. The authors provide titrations on Western blots, but for the p-Upf1 detection, most of the bands have intensities at or below the bottom of the titration range. Given the importance of the Western quantification to the paper, more convincing evidence that the quantified signals are in the linear range of the assay is needed.

*Our response: The Reviewer is correct that some lanes in the Western blot shown in Fig. 1d for FMRP and UPF1, in Fig. 2d for p-UFP1 and GAPDH, and in Fig. 2g for UPF1, are out of the linear range of analysis. Since these western blots are representative of multiple independent analyses, we already had blots that can be substituted for the offending blots. These blots are now used as replacements, and our conclusions do not change.*

4. Do the cultured neurons used for the experiments in Figure 1i-j have elevated p-Upf1?

*Our response: We believe this Reviewer is asking if the cultured neurons developed from P1 cortex of Fmr1-KO mice have an elevated level of p-UPF1 relative to the cultured neurons developed from P1 cortex of WT mice. The answer is yes, and we now include these data as new Fig. S1d. On page 5, lines 109-113, we modified “RNA-seq of cortical neurons deriving from 2-day cultures of P1 cortex (Fig. 1h) revealed that the levels of NMD targets defined here (Fig. 1a and Additional file 1: Table S1) were generally and significantly (P = 8.7 x 10-14) decreased in Fmr1-KO relative to WT mice (Fig. 1h and i).” by adding the following. “concomitantly with an increased abundance of p-UPF1 (Additional file 2: Fig. S1d).”*

Minor points:

1. Line 48--quantification of the mean enrichment would be more informative than the p value (since no info is given regarding the tests used, sample sizes, or comparisons made).

*Our response: We agree with this reviewer’s point. We have changed “P value” to “enrichment” and added the description “(e.g., there is an average ~2-fold enrichment of FMRP with NMD targets relative to other cellular mRNAs)” at the end of the sentence on page 2, lines 48-50.*

2. Figure S1: I'm not sure how to evaluate the statistic that > 50% of the NMD targets are expressed in cerebral cortex and ~40% in the hippocampus. Are these tissue-specific mRNAs or broadly expressed?

*Our response: The Reviewer is referring to the statement on page 3-4, lines 79-84 that reads as follows. “In particular, >50% (527/1027) of NMD targets produce proteins in the cerebral cortex, which regulates motor function and aspects of cognition that include attention and language. The functional significance that 421 NMD targets are expressed in hippocampus (Additional file 2: Fig. S1a) is exemplified by the finding that inhibiting NMD after a prolonged seizure using the small molecule NMDI-14 reduced subsequent spontaneous seizures [12].” We have now modified these sentences as follows. “In particular, >50% (527/1027) of NMD targets defined for N2A cells produce proteins found in the cerebral cortex, which regulates motor function and aspects of cognition that include attention and language. The functional significance that 421 NMD targets defined for N2A cells are expressed in hippocampus (Additional file 2: Fig. S1a) is exemplified by the finding that inhibiting NMD after a prolonged seizure using the small molecule NMDI-14 reduced subsequent spontaneous seizures [12]. Among them, 407 of the 421 NMD targets expressed in hippocampus are also expressed in cerebral cortex. (Additional file 2: Fig. S1a).”*

3. Figure S1: The results of GO analyses are strongly dependent on the background set of genes used to evaluate the null hypothesis, so this information should be included in the methods.

*Our response: We have now added statistics to the GO analyses in Table S2. We have now also inserted a reference to Table S2 on page 4, line 90 and also page 5, line 117.*

4. Abstract: The basis for the claim that the "findings identify the importance of NMD-encoded proteins to brain development" is not clear from the data shown. The previous study used NMD inhibition to rescue neuronal differentiation, but no functional experiments establishing the role of NMD in mouse brain development are included here.

*Our response: What we mean by this statement is that mRNAs identified as NMD targets in this study are expressed throughout the mouse brain (see Fig. S1a) and, based on what proteins they encode (Fig. S1b), are critical for brain development. Given that the Abstract is limited to few words, that we refer Fig. S1a and Fig. S1b and other relevant figures in ways that justify the Abstract, as does the Discussion, we hope that changing the Abstract from “Our findings identify the importance of NMD-encoded proteins to brain development” to “Our findings demonstrate that NMD regulates many neuronal mRNAs that are important for mouse brain development,” is clearer and will encourage readers to read more. Regarding functional experiments, we are gearing up to assay the molecular and behavioral effects of NMD inhibitors using mice now.*

**Second round of review**

**Reviewer 1**

The authors have addressed my previous concerns. i do not have further comments.

**Reviewer 2**

The revised manuscript from Maquat and colleages is improved in several important respects. Most importantly, the RT-PCR data provided in Figure S1C helps to address the question of NMD efficiency in FMRP knockout mouse brains.

Major comments:

1. From the authors' response and the unchanged graphs in Figures 1 and 2, it appears that the blots in Fig. 1d, 2d, and 2g shown in the first version of the paper were still used in the quantification analyses, even though they are "out of the linear range of analysis." Can the authors clarify this point?

Minor comments:

1. Fig. S1d. Quantification of total and p-UPF1 levels should be performed to substantiate the claim of increased phosphorylation in the cultured neurons.

2. It is difficult to see the colocalization of UPF1 and FMRP in cortical neurons in Fig. S2e. Quantification of protein colocalization would be very helpful in this case.

**Authors Response**

**Point-by-point responses to the reviewers’ comments:**

We have also responded to comments made by Referee 2 and yourself.

1. Referee 2 notes that some of the blots are composites from separate blots, and we would request that you address this point. We also require that you provide full, uncropped westerns in the supplemental files.

*Our response: We used different blots, each of which derived biological replicate experiments, for quantitations. We did not mix-and-match different blots, i.e. composite blots. This becomes clear when one examines the uncropped westerns that we included in the previous submission and continue to include in this version.*

2. From the authors' response and the unchanged graphs in Figures 1 and 2, it appears that the blots in Fig. 1d, 2d, and 2g shown in the first version of the paper were still used in the quantification analyses, even though they are "out of the linear range of analysis." Can the authors clarify this point?

*Our response: This reviewer is correct that not all blots in Fig. 1d, 2d and 2g needed to be changed. Those that were changed in the previous revision appeared to be out of the linear range of analysis and consisted of: Fig. 1d, FMRP and UPF1 blots; Fig. 2d, p-UPF1 and GAPDH blots; and Fig. 2g, UPF1 blot. This reviewer is also correct that our graphs in Figures 1 and 2 did not change – our data remain the same since the blots that looked like they were out of the linear range of analysis, when rerun, proved to provide reliable quantitative analyses.*

3. Fig. S1d. Quantification of total and p-UPF1 levels should be performed to substantiate the claim of increased phosphorylation in the cultured neurons.

*Our response: We previously included an analysis of p-UPF1 levels, and for this second revision, we now include the requested analysis of UPF1 levels. Results indicate that the ratio of p-UPF1 to UPF1 was ~10-fold increased in FMR1-KO cells relative to WT cells. This is consistent with our conclusion that p-UPF1 is abnormally upregulated in FMR1-KO neurons.*

4. It is difficult to see the colocalization of UPF1 and FMRP in cortical neurons in Fig. S2e. Quantification of protein colocalization would be very helpful in this case.

*Our response: The dual-channel (488nm/568nm) image shows extensive yellow signals (see magnified regions) that correspond to colocalized signals from the green (FMRP-labelling) and red (UPF1 or pUPF1-labelling) channels. While quantification of fluorescence intensity and signal overlap would not provide meaningful information regarding the protein quantity (without carefully controlling for quenching, fading, and the linear range of signal), we have now performed one-to-one pixel matching using Image J coloc2 plugin software (now noted on page 15 in Methods) to determine the degree of spatial colocalization between FMRP- and UPF1/p-UPF1 labeling. The revised Fig. S2e now includes Pearson’s correlation coefficients for FMRP and UPF1 (r = 0.70) and FMRP and p-UPF1 (r = 0.79) in the merged panel. These calculations demonstrate a high degree of colocalization (Pearson’s correlation coefficient > 0.5) for FMRP and UPF1 and for FMRP and p-UPF1 in each cell.*
